# The Cooperative Roles of Two Kinetoplastid-Specific Kinesins in Cytokinesis and in Maintaining Cell Morphology in Bloodstream Trypanosomes

**DOI:** 10.1371/journal.pone.0073869

**Published:** 2013-09-12

**Authors:** Ying Wei, Huiqing Hu, Zhao-Rong Lun, Ziyin Li

**Affiliations:** 1 State Key Laboratory of Biocontrol, School of Life Sciences, Sun Yat-Sen University, Guangzhou, Guangdong, China; 2 Department of Microbiology and Molecular Genetics, University of Texas Medical School, Houston, Texas, United States of America; Instituto Butantan, Laboratório Especial de Toxinologia Aplicada, Brazil

## Abstract

The cytoskeleton of *Trypanosoma brucei*, a unicellular eukaryote and a parasitic protozoan, is defined by the subpellicular microtubule corset that is arranged underneath the plasma membrane. We recently identified two orphan kinesins, TbKIN-C and TbKIN-D, that cooperate to regulate the organization of the subpellicular microtubule corset and thereby maintain cell morphology in the procyclic form of *T. brucei*. In this report, we characterize the function of TbKIN-C and TbKIN-D in the bloodstream form of *T. brucei* and investigate their functional cooperation in both the bloodstream and procyclic forms. TbKIN-C and TbKIN-D form a tight complex *in vivo* in the bloodstream form. TbKIN-C is strongly enriched at the posterior tip of the cell, whereas TbKIN-D is distributed throughout the cell body at all cell cycle stages. RNAi of TbKIN-C or TbKIN-D in the bloodstream form inhibits cell proliferation and leads to cell death, due to cytokinesis defects. RNAi of TbKIN-C and TbKIN-D also results in defects in basal body segregation, but does not affect the synthesis and segregation of the flagellum and the flagellum attachment zone (FAZ) filament. Knockdown of TbKIN-C and TbKIN-D does not disrupt the organization of the subpellicular microtubule corset, but produces multinucleated cells with an enlarged flagellar pocket and misplaced flagella. Interestingly, depletion of TbKIN-C results in rapid degradation of TbKIN-D and, similarly, knockdown of TbKIN-C destabilizes TbKIN-D, suggesting that formation of TbKIN-C/TbKIN-D complex stabilizes both kinesins and is required for the two kinesins to execute their essential cellular functions. Altogether, our results demonstrate the essential role of the two kinesins in cell morphogenesis and cytokinesis in the bloodstream form and the requirement of heteromeric complex formation for maintaining the stability of the two kinesins.

## Introduction

*Trypanosoma brucei* is an early-branched unicellular eukaryote and the causative agent of human sleeping sickness and nagana in animals in Sub-Sahara region of Africa. A trypanosome cell contains a single copy of organelles/cytoskeletal structures such as flagellum, basal body, nucleus, mitochondrion, and Golgi, and each of these organelles/cytoskeletal structures is duplicated and segregated into the two daughter cells during the cell division cycle. The single-copy organelles are organized at distinct positions in the cytoskeleton that is represented by an array of subpellicular microtubules arranged underneath the plasma membrane [1]. These subpellicular microtubules possess an intrinsic polarity and are cross-linked to each other and to the plasma membrane, forming a cage-like structure with all the organelles situated at their respective locations [2], [3]. The microtubule cytoskeleton of *T. brucei* has been demonstrated to be essential for maintenance of cell morphology and for segregation of organelles during cell division.

In addition to the microtubule cytoskeleton, the flagellum in a trypanosome cell is also essential for maintaining cell morphology [4], [5], [6]. It contains a canonical 9+2 microtubule axoneme and is attached to the cell body via a unique cytoskeletal structure, the flagellum attachment zone (FAZ) [2], which consists of a single protein filament and a specialized set of four microtubules [7]. The elongation of FAZ appears to drive the segregation of basal bodies [8], [9], which are known to constitute the cell’s microtubule organizing centers (MTOCs) that nucleate flagellum and are linked to the kinetoplast, the cell’s unique mitochondrial DNA network [1]. Replication and segregation of the multiple single-copy organelles during the cell cycle are well coordinated with the growth of the new flagellum and the new FAZ [10], [11], [12], and the length and position of the flagellum appear to define the cleavage furrow that impacts precise cytokinesis [4].

Despite the tremendous efforts leading to our understanding of the structure and function of the cytoskeleton and flagellum in the procyclic form, our knowledge about the microtubule cytoskeleton and flagellum in the bloodstream form of *T. brucei* is limited. The cell morphology of the two life cycle forms differs slightly. For example, the procyclic-form cell contains a flagellar connector that anchors the new flagellum to the old flagellum, whereas the flagellar connector appears to be absent in the bloodstream-form cell [13]. In the procyclic-form cell, one daughter kinetoplast sits between the two segregated nuclei, but in the bloodstream-form cell both daughter kinetoplasts are located posterior to the two nuclei and are subject to a limited movement during their segregation. However, it is not clear whether these morphological differences contribute to any cell biological distinctions between the two forms, such as the distinctions in cell cycle regulation and cell motility. The two life cycle forms appear to respond differently to defects in cell cycle and cell motility. Any mitotic defects in the procyclic form generally do not inhibit cytokinesis, whereas the same defect in the bloodstream form completely arrests cytokinesis but does not inhibit the next mitotic cycle (for a review, see [14]). Defects in cell motility do not inhibit cell proliferation in the procyclic form, but significantly inhibit cell proliferation and lead to cell death in the bloodstream form [5].

We previously identified two trypanosome-specific orphan kinesins, TbKIN-C and TbKIN-D, that cooperate to maintain cell morphology by regulating the organization of the subpellicular microtubule corset in the procyclic form [15], [16]. Both kinesins associate with the microtubule cytoskeleton, but TbKIN-C is additionally enriched at the posterior tip of the cell. RNAi of TbKIN-C and TbKIN-D results in massive accumulation of cytoplasmic microtubules and disorganization of the subpellicular microtubule corset, which leads to elongation of the posterior portion and round-up of the middle portion of the cell [15], [16]. Although the precise role of TbKIN-C and TbKIN-D in maintaining cell morphology in the procyclic form remains elusive, it is believed that the two kinesins regulate the dynamics of microtubules through an unknown mechanism.

In this paper, we report the functional characterization of TbKIN-C and TbKIN-D in the bloodstream form of *T. brucei*. TbKIN-D is distributed to the entire cell body throughout the cell cycle, whereas TbKIN-C is enriched at the posterior tip of the cell, with additional distribution to the cell body. RNAi of TbKIN-C or TbKIN-D in the bloodstream form resulted in defects in basal body segregation and cytokinesis, but did not compromise the organization of the subpellicular microtubule corset as demonstrated in the procyclic form [15], [16]. Moreover, RNAi of the two kinesins in the bloodstream form appeared to disrupt flagellar pocket morphogenesis, resulting in an enlarged flagellar pocket and misplaced flagella. Such defects were, however, not observed in the procyclic form depleted of the two kinesins [15], [16]. We also found that depletion of one kinesin from the cell resulted in the degradation of the other kinesin in both the procyclic and bloodstream forms, suggesting the formation of heteromeric TbKIN-C/TbKIN-D complex is required for stabilization of the two kinesins.

## Results

### TbKINC and TbKIN-D Form a Complex in vivo in the Bloodstream Form

**Figure 1:**
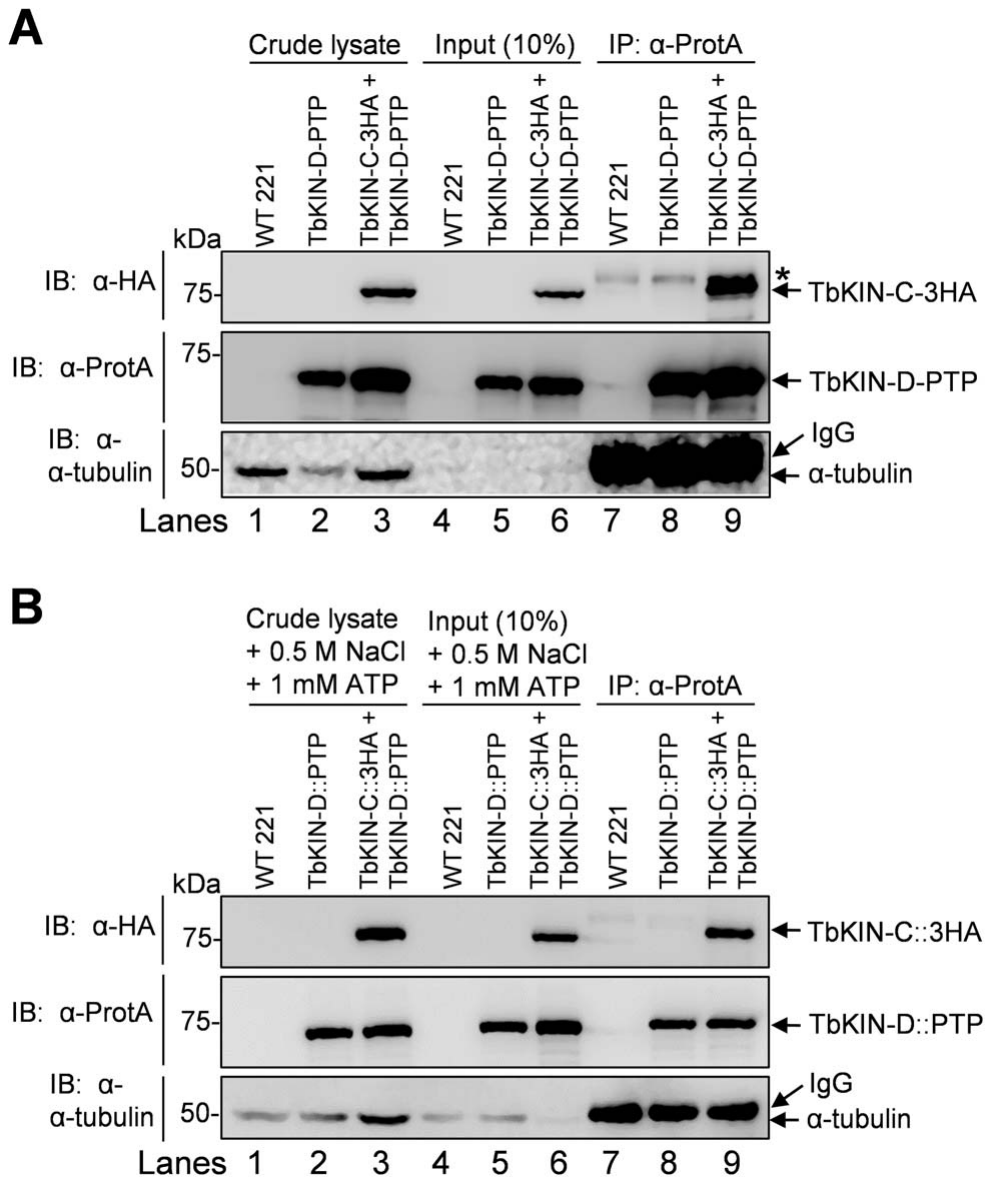
TbKIN-C and TbKIN-D form a complex *in vivo* in the bloodstream form. (**A**). Co-immunoprecipitation to detect the *in vivo* interaction between TbKIN-C and TbKIN-D in the bloodstream form of *T. brucei*. TbKIN-D-PTP and TbKIN-C-3HA were co-expressed from their respective endogenous locus in wild-type 221 cell line. Co-immunoprecipitation was carried out by incubating the cleared cell lysate with IgG sepharose beads and subsequent immunoblotting of immunoprecipitates with anti-HA antibody, anti-Protein A antibody, and anti-tubulin antibody, respectively. The asterisk indicates a non-specific band recognized by the anti-HA antibody. (**B**). The *in vivo* interaction between TbKIN-C and TbKIN-D is not bridged by microtubules. Crude cell lysates were incubated with 1 mM ATP and 500 mM NaCl to dissociate kinesin proteins from microtubules, and then cleared by centrifugation before immunoprecipitation. Co-immunoprecipitation was carried out as in (**A**). Lanes 1–3: crude cell lysate: boiled cell lysate without centrifugation; Lanes 4–6: Input: centrifugation-cleared cell lysate that was used for immunoprecipitation; Lanes 7–9: immunoprecipitation. The higher molecular mass band detected by anti-tubulin antibody in lanes 7–9 was the IgG heavy chain, which is slightly bigger than tubulins (50 kDa).

To investigate whether TbKIN-C and TbKIN-D interact with each other *in vivo* in bloodstream cells, we generated a cell line expressing PTP-tagged TbKIN-D and triple HA-tagged TbKIN-C from their respective endogenous locus. Since both TbKIN-C and TbKIN-D associate with the microtubule cytoskeleton, it is possible that the interaction between the two kinesins is bridged by microtubules. To rule out this possibility, we cleared the cell lysate by centrifugation to remove microtubules before immunoprecipitation. As shown by western blot with anti-tubulin antibody, no tubulin was detected in the cleared lysate used for co-immunoprecipitation (Figure 1A, Lanes 4–6). Co-immunoprecipitation showed that precipitation of TbKIN-D-PTP was capable of pulling down TbKIN-C-3HA from trypanosome cell lysate that was cleared by centrifugation (Fig. 1A).

To further confirm that the interaction between TbKIN-C and TbKIN-D is not bridged by microtubules, we carried out co-immunoprecipitation experiments by adding 1 mM ATP and 500 mM NaCl to the cell lysate to dissociate kinesin-microtubule interactions. The cell lysate was cleared by centrifugation before immunoprecipitation. Treatment of trypanosome cells with high salt is known to disassemble the cytoskeletal microtubule corset [17], and indeed a small amount of tubulin proteins was detected in the cleared lysate (Fig. 1B, Lanes 4–6). In the absence of microtubules (the higher molecular mass band detected by anti-tubulin antibody in lanes 7–9 of Fig. 1A and 1B represents IgG heavy chain), TbKIN-C-3HA was still co-precipitated with TbKIN-D-PTP (Fig. 1B). The results presented in Figure 1A and 1B indicate that TbKIN-C and TbKIN-D form a complex in the bloodstream form, which is not bridged by cytoskeletal microtubules. The results also suggest that the two kinesins likely function together in the bloodstream form.

### Subcellular Localization of TbKIN-C and TbKIN-D in the Bloodstream Form

**Figure 2:**
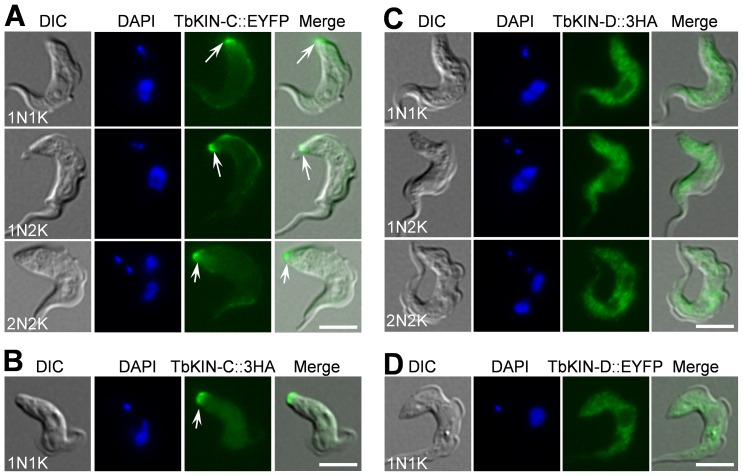
Subcellular localization of TbKIN-C and TbKIN-D in the bloodstream form. TbKIN-C and TbKIN-D were each tagged with EYFP or triple HA epitope at the C-terminus in their endogenous locus. Localization of EYFP-tagged proteins was examined directly under a fluorescence microscope, whereas localization of 3HA-tagged proteins were monitored after immunostaining the cells with FITC-conjugated anti-HA antibody. Cells were fixed with paraformaldehyde. Arrows indicate the enrichment of TbKIN-C at the posterior tip of the cells. Bars: 5 µm.

In the procyclic form, TbKIN-C is enriched at the posterior tip of the cells in addition to being spread out throughout the cytoskeleton [16], whereas TbKIN-D is localized to the entire cytoskeleton throughout the cell cycle [15]. To determine the subcellular localization of TbKIN-C and TbKIN-D in the bloodstream-form cells, we generated the cell lines expressing either EYFP-tagged or 3HA-tagged TbKIN-C and TbKIN-D and found that tagging with either EYFP or the triple HA epitope exhibited identical subcellular localization (Fig. 2), suggesting that tagging with EYFP and HA epitope does not interfere with the subcellular localization of TbKIN-C and TbKIN-D. TbKIN-C was found strongly enriched at the posterior tip of the cells at all cell cycle stages (Fig. 2A, B, arrows). In addition, it also appeared to spread throughout the cell body, albeit the TbKIN-C fluorescence signal in the cell body was less intense than the fluorescence signal at the posterior tip (Fig. 2A, B). TbKIN-D was not enriched at the posterior tip, but was distributed throughout the cell body (Fig. 2C, D). The localization patterns of both proteins in the bloodstream form are almost identical to that in the procyclic form [15], [16].

Since TbKIN-C and TbKIN-D interact *in vivo* in trypanosomes (Fig. 1), the lack of complete co-localization between the two kinesins suggests that only a fraction of TbKIN-C and TbKIN-D proteins form a complex. The co-localization of TbKIN-C and TbKIN-D could occur either at the posterior tip of the cell, where TbKIN-C level is much higher than TbKIN-D level, or in the other part of the cell body, where TbKIN-D level is much higher than TbKIN-C level, or at both locations (Fig. 2). Nevertheless, the enrichment of TbKIN-C and TbKIN-D at distinct subcellular locations also suggests that the two kinesins may play additional roles that are not shared by their partners.

### RNAi of TbKIN-C and TbKIN-D in the Bloodstream Form Inhibits Cell Proliferation

**Figure 3:**
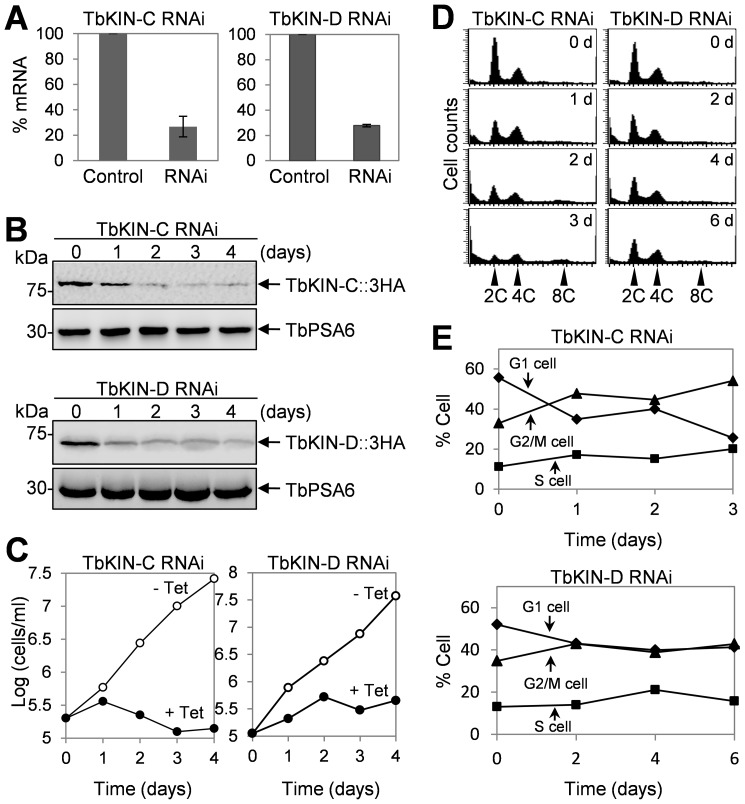
RNAi of TbKIN-C and TbKIN-D in the bloodstream form inhibits cell proliferation. (**A**). TbKIN-C and TbKIN-D mRNA level in control and RNAi cells detected by quantitative RT-PCR. (**B**). TbKIN-C and TbKIN-D protein level in control and RNAi cells detected by Western blot with anti-HA antibody. (**C**). RNAi of TbKIN-C and TbKIN-D resulted in significant growth inhibition. (**D**). Flow cytometry analysis of TbKIN-C and TbKIN-D RNAi cells. (**E**). Quantitative analysis of the flow cytometry experiments from panel D, showing the percentage of G1, S-phase, and G2/M cells upon RNAi induction of TbKIN-C and TbKIN-D.

To understand the functions of TbKIN-C and TbKIN-D in the bloodstream form, RNAi was carried out. Three clonal cell lines harboring the RNAi constructs targeting TbKIN-C or TbKIN-D were obtained, and our preliminary analysis showed that all three monoclonal cell lines exhibit almost identical cell growth defects. Therefore, only one clonal cell line was characterized in detail. Quantitative RT-PCR showed that upon RNAi induction for 2 days levels of TbKIN-C and TbKIN-D mRNA were reduced to about 25% of their respective mRNA level in the uninduced control cells (Fig. 3A). To monitor the protein level of TbKIN-C and TbKIN-D after RNAi induction, we tagged the endogenous TbKIN-C and TbKIN-D with a triple HA epitope in their respective RNAi cell lines. Western blot showed that TbKIN-C protein was decreased to about half of the protein level in control cells after RNAi induction for 1 day and was significantly down-regulated after 2 days of RNAi induction, whereas TbKIN-D protein was significantly reduced after RNAi for only 1 day (Fig. 3B). However, neither TbKIN-C nor TbKIN-D was completely depleted from the cells up to RNAi induction for 4 days (Fig. 3B). Nevertheless, the significant reduction in TbKIN-C protein level resulted in severe growth inhibition and cell death after 4 days (Fig. 3C), indicating that TbKIN-C is indispensable for cell proliferation and cell viability in the bloodstream form. Decrease of TbKIN-D protein also significantly slowed down cell proliferation but did not kill the cells even after RNAi induction for up to 6 days (Fig. 3C and data not shown). This result suggests that TbKIN-D is also essential for cell proliferation in the bloodstream form.

To investigate whether the reduced growth rate of TbKIN-C and TbKIN-D RNAi cells was attributed to any defects in the cell cycle, we carried out flow cytometry assays. We found that RNAi of TbKIN-C and TbKIN-D each led to gradual decrease of cells with 2C DNA content, which was accompanied by an increase of cells with 4C DNA content from ∼33% to ∼55% after RNAi of TbKIN-C for 3 days and from ∼34% to ∼43% after RNAi of TbKIN-D for 6 days (Fig. 3D, E). Additionally, a small 8C DNA peak in the flow cytometry histogram was detected in TbKIN-C RNAi cells after RNAi induction for 3 days, but the 8C DNA peak in TbKIN-D RNAi cells was not readily detectable (Fig. 3D). The results suggest that RNAi of TbKIN-C and TbKIN-D inhibits cytokinesis in the bloodstream form, but TbKIN-C RNAi appears to exert a stronger effect on cell cycle than TbKIN-D RNAi.

**Figure 4:**
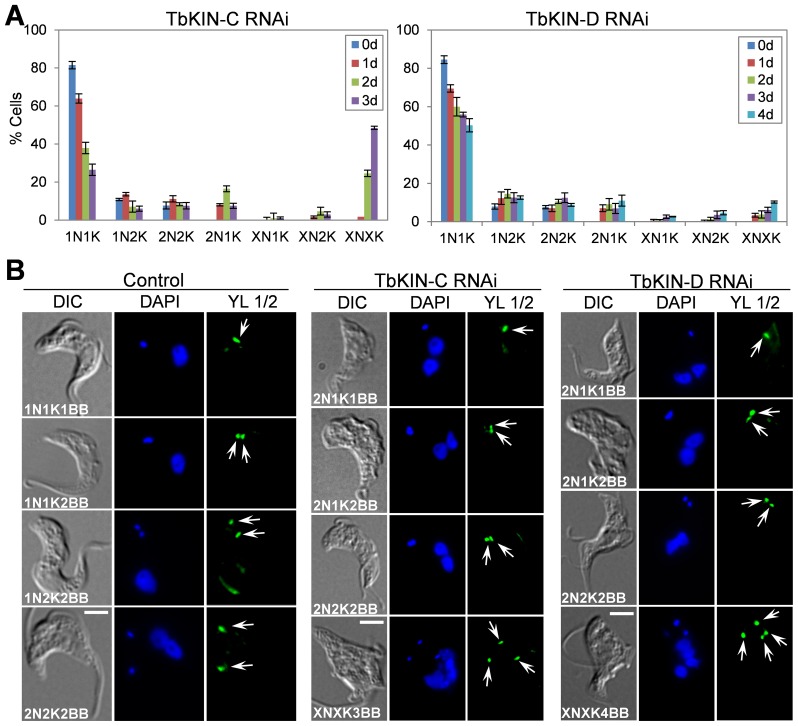
RNAi of TbKIN-C and TbKIN-D disrupts basal body segregation in the bloodstream form. (**A**). Tabulation of cells with different numbers of nucleus (N) and kinetoplast (K) upon TbKIN-C and TbKIN-D knockdown. X>2. (**B**). Effect of TbKIN-C and TbKIN-D RNAi on basal body replication and segregation. Mature basal bodies (arrows) were stained by YL 1/2 antibody. Bars: 5 µm.

To further characterize the effect of TbKIN-C and TbKIN-D RNAi on the nuclear and kinetoplast cycles, we stained the cells with DAPI for nuclear and kinetoplast DNA and counted the number of cells with different numbers of nucleus and kinetoplast. In TbKIN-C RNAi cells, there was a drastic decrease of cells with one nucleus and one kinetoplast (1N1K) from ∼80% to ∼25% (Fig. 4A). The number of 1N2K cells was slightly decreased, but the number of 2N2K cells remained constant during the 3 days of RNAi induction (Fig. 4A). Some unusual cell types, such as 2N1K cells, emerged to ∼16% of the total cell population after RNAi for 2 days and then declined (Fig. 4A). Moreover, there was a significant increase of cells with multiple nuclei and multiple kinetoplasts (XNXK), which accumulated to ∼50% of the cell population after RNAi induction for 3 days (Fig. 4A), further supporting the notion that cytokinesis was inhibited in TbKIN-C RNAi cells. Unlike TbKIN-C RNAi, however, TbKIN-D RNAi appeared to exert a less severe effect on the nuclear and kinetoplast cycles, which agrees with the less severe defect in cell growth and cell cycle progression (Fig. 3C–E). Upon TbKIN-D RNAi induction for up to 4 days, the number of 1N1K cells decreased from ∼80% to ∼50%, whereas the numbers of 1N2K and 2N2K cells slightly increased up to ∼5–6% (Fig. 4A). Moreover, like TbKIN-C RNAi, 2N1K cells and XNXK cells also emerged to about 10% of the total cell population, but it was significantly less than that in TbKIN-C RNAi cells (Fig. 4A). Together, these results suggest that both TbKIN-C and TbKIN-D are required for kinetoplast segregation and cell division but not nuclear division in the bloodstream form.

### RNAi of TbKIN-C and TbKIN-D Inhibits Basal Body Segregation in the Bloodstream Form

The emergence of 2N1K cells suggests the defect in kinetoplast segregation in TbKIN-C and TbKIN-D RNAi cells (Fig. 4A). It is known that basal body duplication and segregation drives the segregation of kinetoplasts in trypanosomes [18], so we asked whether the defect in kinetoplast segregation in TbKIN-C and TbKIN-D RNAi cells was attributed to impaired basal body replication and/or segregation. To detect the basal bodies we performed immunofluorescence microscopy using the YL 1/2 antibody, which specifically stain the mature basal bodies as well as tyrosinated α-tubulin in trypanosomes [19]. In the control bloodstream-form cells, the 1N1K cells contained either a single basal body, which represented the G1 cells, or two closely associated basal bodies that were just duplicated, which were frequently found in S-phase cells (Fig. 4B). Without exception, all the control 1N2K and 2N2K cells possessed two basal bodies (Fig. 4B). Unlike in the procyclic form where the two basal bodies in 2N2K cells were well separated up to about 7 µm, with one basal body positioned in the middle of the two nuclei, the two basal bodies in the bloodstream 2N2K cells were both posterior to both nuclei and were only separated up to about 3–4 µm (Fig. 4B). However, the two basal bodies in the 2N2K cells deficient in TbKIN-C or TbKIN-D appeared to be only slightly separated, with an average inter-basal body distance of about 0.5 µm (Fig. 4B, arrows). Additionally, the 2N1K cells accumulated in TbKIN-C and TbKIN-D RNAi cells contained either a single basal body or two basal bodies that were very closely to each other (Fig. 4B, arrows), and the XNXK cells all contained multiple basal bodies that were all posterior to the multiple nuclei (Fig. 4B, arrows). These results suggest that segregation, but not replication, of basal bodies was impaired in TbKIN-C and TbKIN-D RNAi cells. Therefore, defects in basal body segregation contribute to the inhibited kinetoplast segregation in the RNAi cells. However, it should be noted that the defect in basal body segregation in TbKIN-C and TbKIN-D RNAi cells is unlikely due to the regulatory role of TbKIN-C and TbKIN-D in basal body segregation, but could be attributed to the distorted cell morphology.

**Figure 5:**
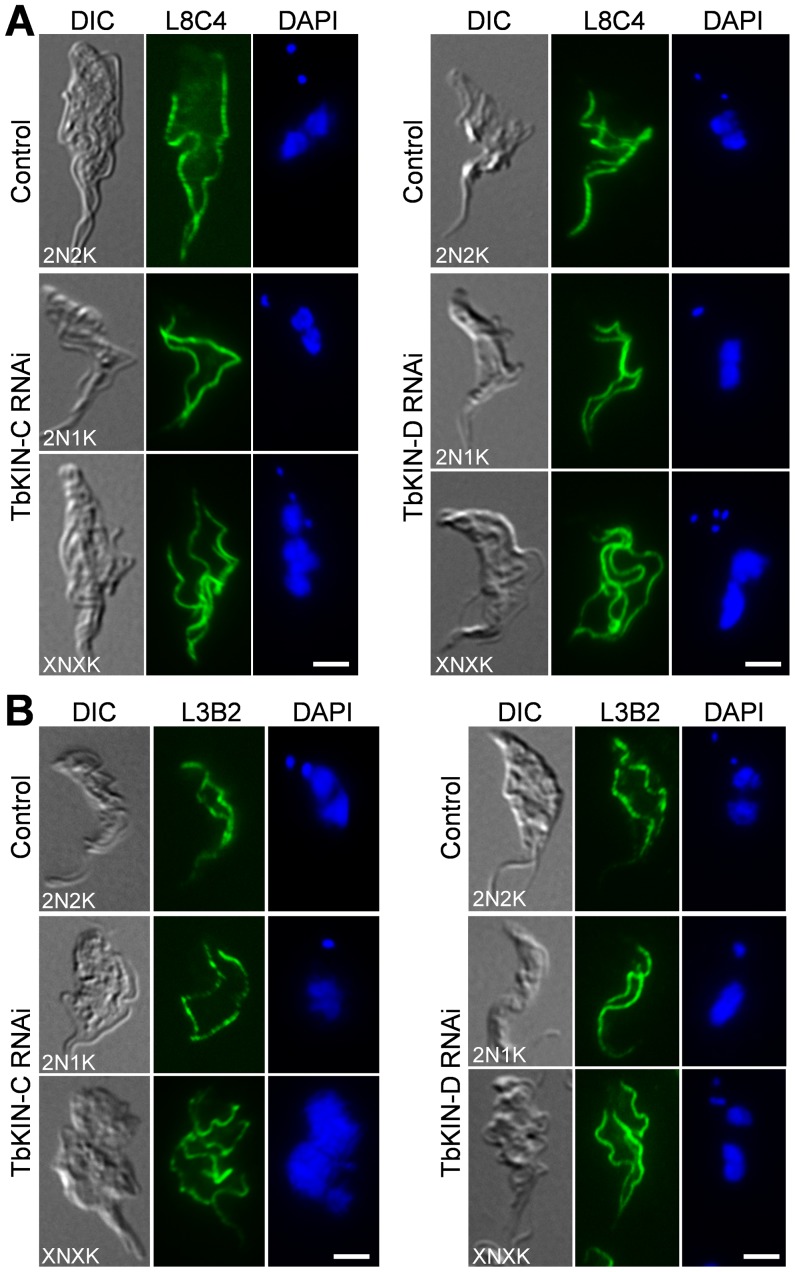
Effect of TbKIN-C and TbKIN-D RNAi on the synthesis and segregation of the flagellum and the FAZ filament in the bloodstream form. (**A**). Effect of TbKIN-C and TbKIN-D RNAi on the flagellum. Cells were immunostained with L8C4 antibody for flagella and counterstained with DAPI for the nuclear and kinetoplast DNA. (**B**). Effect of TbKIN-C and TbKIN-D RNAi on the FAZ filament. Cells were immunostained with L3B2 antibody for the FAZ and counterstained with DAPI for the nuclear and kinetoplast DNA. Bars: 5 µm.

We next examined whether RNAi of TbKIN-C and TbKIN-D affects the synthesis and segregation of flagella and the FAZ filament. RNAi was induced for 2 days for TbKIN-C and 3 days for TbKIn-D. Cells were immunostained with L8C4 antibody, which labels a protein in the paraflagellar rod of the flagellum in trypanosomes [7], and with L3B2 antibody, which labels the FAZ1 protein in the FAZ filament [7], [9]. We found that all the 2N1K cells produced by RNAi of TbKIN-C or TbKIN-D contained two full-length flagella and two full-length FAZ filaments and all the multi-nucleated (>2N) cells contained multiple (>2) flagella and FAZ filaments (Fig. 5). These observations suggest that RNAi of either kinesins did not disrupt the synthesis and segregation of flagella and the FAZ filaments, similar to the resulted obtained in the procyclic form reported previously [15], [16].

### Effect of TbKIN-C and TbKIN-D RNAi on the Subpellicular Microtubule Corset

**Figure 6:**
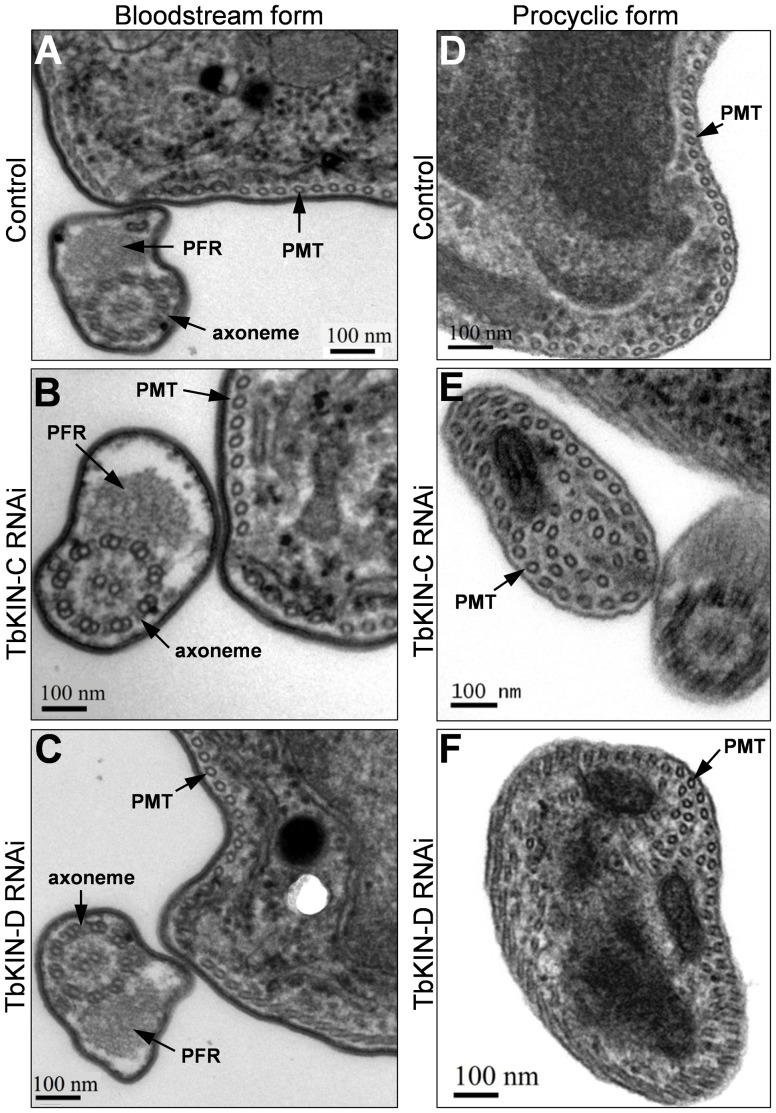
Effect of TbKIN-C and TbKIN-D RNAi on the subpellicular microtubule corset in the bloodstream and procyclic forms. (**A–C**). Thin sections of a control cell (panel **A**), a TbKIN-C RNAi cell (panel **B**), and a TbKIN-D RNAi cell (panel **C**) of the bloodstream form. The subpellicular microtubule corset (PMT), the paraflagellar rod (PFR), and the flagellar axoneme are indicated. Bars: 100 nm. (**D–F**). Thin sections of a control cell (panel **D**), a TbKIN-C RNAi cell (panel **E**), and a TbKIN-D RNAi cell (panel **E**) of the procyclic form. Additional cytoplasmic microtubules are detected in addition to the subpellicular microtubule corset (PMT) that is underneath the membrane. Bars: 100 nm.

Unlike in the procyclic-form cells in which depletion of TbKIN-C and TbKIN-D each resulted in massive accumulation of tyrosinated (i.e., newly assembled) microtubules at the posterior portion of the cell [15], [16], RNAi of the two genes in the bloodstream form did not lead to accumulation of tyrosinated microtubules, as stained by the YL 1/2 antibody (Fig. 4B). The accumulation of newly assembled microtubules in the procyclic form appears to be contributed by *de novo* assembly of cytoplasmic microtubules [15], [16]. Moreover, since RNAi of the two kinesin genes in the procyclic form also disrupted the organization of the subpellicular microtubule corset [15], [16], we further examined the bloodstream-form RNAi cells by transmission electron microscopy. The subpellicular microtubule corset in TbKIN-C and TbKIN-D RNAi cells of the bloodstream form appeared to be unaltered, with the individual microtubules evenly spaced and arranged underneath the plasma membrane (Fig. 6A–C). Additionally, among the many (>200) thin sections examined, no cytoplasmic microtubules were detected in the two bloodstream-form RNAi cells (Fig. 6A–C), which was in contrast to the procyclic cells depleted of TbKIN-C and TbKIN-D in which numerous cytoplasmic microtubules were found in most thin sections examined (Fig. 6D–F, also see [15], [16]). Taken together, these results indicate that RNAi of TbKIN-C and TbKIN-D in the bloodstream form does not affect the organization of the subpellicular microtubule corset and does not lead to *de novo* assembly of cytoplasmic microtubules. The mechanisms behind the distinct phenotypes between the two life cycle forms are not clear.

**Figure 7:**
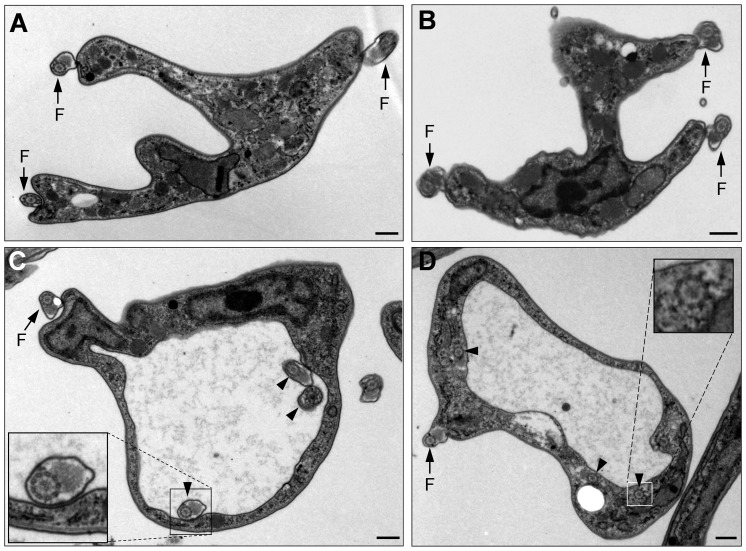
Electron microscopic analysis of TbKIN-C and TbKIN-D RNAi cells of the bloodstream form. (**A, B**). A multi-nucleated TbKIN-C RNAi cell (**A**) and TbKIN-D RNAi cell (**B**). Arrows indicated the three flagella attached to the cell body. (**C, D**). TbKIN-C RNAi cells with an extremely large flagellar pocket. The arrow indicated the flagellum that has exited the flagellar pocket. The arrowheads in panel **C** pointed to the flagellar axoneme with a normal PFR inside the enlarged flagellar pocket, whereas the arrowheads in panel **D** pointed to the flagellar axoneme-like structure in the cytoplasm near the flagellar pocket. Insets are the enlarged view of the flagellum (panel **C**) and the flagellar axoneme-like structure. Bars: 0.4 µm.

Among the many (>200) thin sections examined, ∼60% of them from TbKIN-C RNAi cells and ∼10% from TbKIN-D RNAi cells contained multiple flagella that were either attached to the cell body (Fig. 7A, B, arrows) or resided inside the flagellar pocket (Fig. 7C, arrowheads). In the latter case, the flagellar pocket became very large and the paraflagellar rod (PFR) was abnormally present in the flagella inside the pocket (Fig. 7C, inset). In normal trypanosome cells, the PFR is only present in the flagellum that has exited the flagellar pocket. The cells with large flagellar pocket constituted ∼8% of the thin sections examined in TbKIN-C RNAi cells and ∼5% in TbKIN-D RNAi cells. Intriguingly, in other cells that also contained a large flagellar pocket, a number of flagellar axonemes, which did not have associated PFR, were detected in the cytoplasm near the enlarged flagellar pocket (Fig. 7D, arrowheads and inset). These observations suggest that flagellar pocket morphogenesis was likely impaired in the RNAi cells, and there likely were also defects in endocytosis in these cells. However, given that only ∼8% of TbKIN-C RNAi cells and ∼5% of TbKIN-D RNAi cells possessed enlarged flagellar pocket, the enlargement of flagellar pocket could attributed to distorted cell morphology.

### Interdependence of TbKIN-C and TbKIN-D for Protein Stability

**Figure 8:**
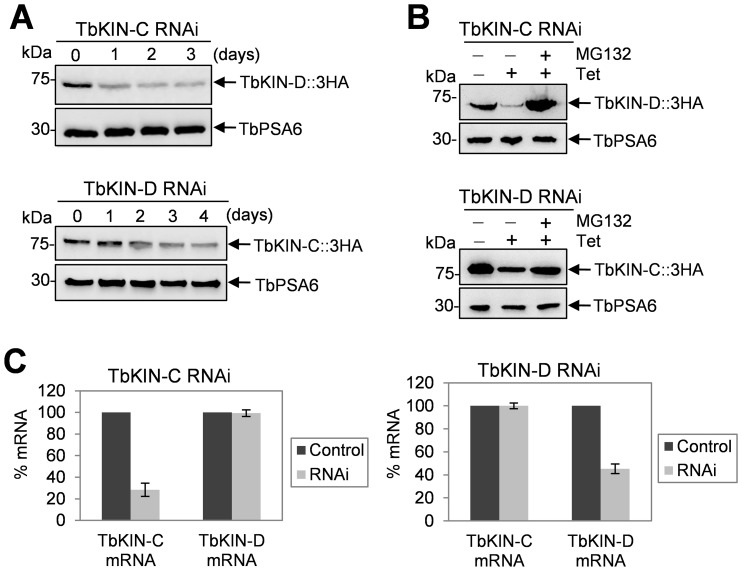
Effect of TbKIN-C RNAi on TbKIN-D protein level and TbKIN-D RNAi on TbKIN-C protein level in the bloodstream form. (**A**). Western blot to detect TbKIN-D protein level in TbKIN-C RNAi cells and TbKIN-C protein level in TbKIN-D RNAi cells in the bloodstream form. (**B**). Western blot to detect TbKIN-D protein level in TbKIN-C RNAi cells and TbKIN-C protein level in TbKIN-D RNAi cells in the procyclic form. MG132 (25 µM) was added to TbKIN-C RNAi cells (day 2) and TbKIN-D RNAi cells (day 3) and incubated for 6 hrs. (**C**). TbKIN-C and TbKIN-D mRNA level in TbKIN-C RNAi cells and TbKIN-D RNAi cells measured by quantitative RT-PCR in the bloodstream form.

Since TbKIN-C and TbKIN-D form a complex in both the procyclic form [15] and the bloodstream form (Fig. 1), we asked whether depletion of one component of the complex affected the abundance of the other. To this end, we tagged the endogenous TbKIN-C in TbKIN-D RNAi cell line and TbKIN-D in TbKIN-C RNAi cell line with a triple HA epitope, and examined the protein level before and after RNAi induction by immunoblotting with anti-HA antibody. In the bloodstream form, we found that TbKIN-C protein level gradually declined upon TbKIN-D RNAi and that TbKIN-D protein level also decreased after TbKIN-C RNAi (Fig. 8A). This observation suggests that RNAi of one kinesin likely resulted in 26S proteasome-mediated degradation of the other kinesin. To test this possibility, we treated the RNAi cells with the proteasome inhibitor MG132, which has been demonstrated to be capable of inhibiting the 26S proteasome activity and proteasome-mediated protein degradation in trypanosomes [20]. In the presence of MG132, TbKIN-C protein was stabilized in the TbKIN-D RNAi cells, and TbKIN-D was not down-regulated by TbKIN-C RNAi (Fig. 8B), suggesting that TbKIN-C protein in TbKIN-D RNAi cells and TbKIN-D protein in TbKIN-C RNAi cells are indeed degraded by the 26S proteasome. However, the down-regulation of TbKIN-C by TbKIN-D RNAi and vice versa could be attributed to off-target effect of RNAi since both *TbKIN-C* and *TbKIN-D* encode kinesin-like proteins. Nevertheless, the gene fragments targeted by RNAi in the two genes do not share high sequence homology (data not shown), thus excluding the possibility that RNAi against TbKIN-C also silenced TbKIN-D and vice versa. To experimentally verify this notion, we performed quantitative RT-PCR assays and found that RNAi only silenced the target genes, with little effect on the other kinesin genes (Fig. 8C), indicating that RNAi against TbKIN-C or TbKIN-D was highly specific.

**Figure 9:**
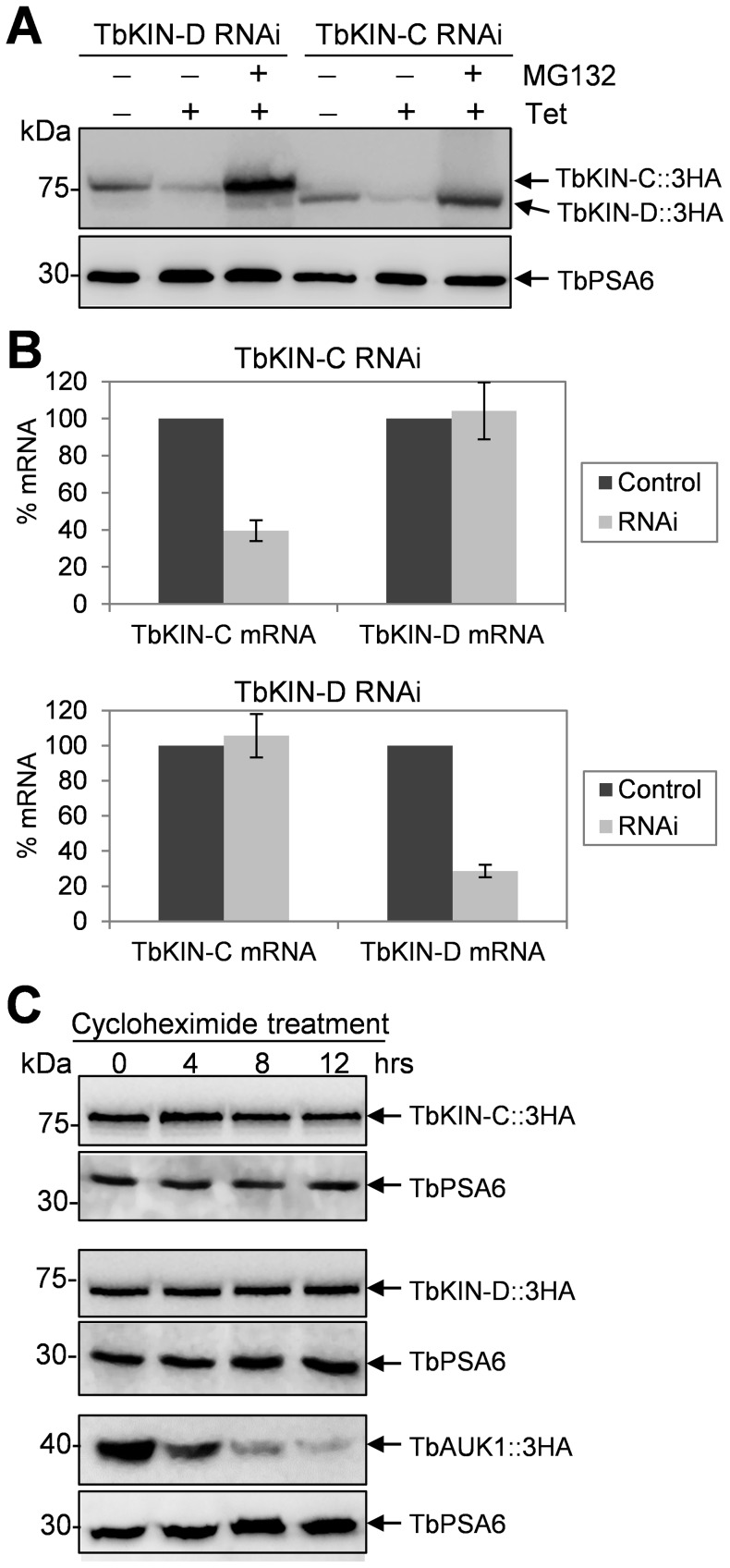
Interdependence of TbKIN-C and TbKIN-D for their stability in the procyclic form. (**A**). Western blot to detect TbKIN-D protein level in TbKIN-C RNAi cells and TbKIN-C protein level in TbKIN-D RNAi cells in the procyclic form. MG132 (50 µM) was added to TbKIN-C RNAi cells and TbKIN-D RNAi cells at day 2 of RNAi induction and incubated for 8 hrs. (**B**). TbKIN-C and TbKIN-D mRNA level in TbKIN-C RNAi cells and TbKIN-D RNAi cells measured by quantitative RT-PCR in the procyclic form. (**C**). Stability of TbKIN-C and TbKIN-D proteins in wild-type procyclic cells. Cells were treated with cycloheximide and time-course samples were immunoblotted with anti-HA antibody to detect the levels of TbKIN-C-3HA and TbKIN-D-3HA proteins. TbAUK1, the Aurora-like kinase in trypanosomes that is known to be degraded by the proteasome, was included as the positive control. Levels of TbPSA6 protein was monitored to serve as the loading control.

We next asked whether TbKIN-C and TbKIN-D are regulated in the same way in the procyclic form. We found that in procyclic cells TbKIN-C protein level was decreased upon TbKIN-D RNAi and TbKIN-D protein level was decreased upon TbKIN-C RNAi (Fig. 9A), which was not due to down-regulation of the mRNA (Fig. 9B) because measurement of the mRNA level of TbKIN-C in TbKIN-D RNAi cells and the mRNA level of TbKIN-D in TbKIN-C RNAi cells did not detect any decrease when compared with the mRNA level in the control cells (Fig. 9B). Moreover, in the presence of MG-132, both TbKIN-C and TbKIN-D proteins were stabilized in the RNAi background (Fig. 8A), indicating that both proteins were degraded by the 26S proteasome when the TbKIN-C/TbKIN-D complex was disrupted upon depletion of any of the two proteins by RNAi from the cells.

Finally, to examine whether TbKIN-C and TbKIN-D are short-lived proteins when they are in the complex, the half-life of both proteins was monitored. Procyclic cells expressing endogenously 3HA tagged TbKIN-C and TbKIN-D were treated with cycloheximide, and time-course samples were collected and immunoblotted with anti-HA antibody. The results showed that both TbKIN-C and TbKIN-D were not destroyed after 12 hrs of cycloheximide treatment (Fig. 9C), suggesting that both proteins were very stable when in the complex. Altogether, these results suggest that formation of the TbKIN-C/TbKIN-D complex stabilizes both TbKIN-C and TbKIN-D and prevents them from degradation by the 26S proteasome.

## Discussion

We previously identified two trypanosome-specific kinesins, TbKIN-C and TbKIN-D, that form a complex and regulate the organization of the subpellicular microtubule corset in the procyclic form of *T. brucei* [15], [16]. RNAi of TbKIN-C and TbKIN-D in the procyclic form resulted in disorganization of the microtubule corset and *de novo* assembly of microtubules in the cytoplasm, which, consequently, led to distorted cell morphology [15], [16]. In the current report, we explored the function of TbKIN-C and TbKIN-D in the bloodstream form of *T. brucei* and found, surprisingly, that RNAi of both kinesins did not disrupt the organization of the subpellicular microtubule corset and did not produce cytoplasmic microtubules in the bloodstream form (Fig. 6). This unexpected finding suggests that TbKIN-C and TbKIN-D are not involved in regulating the dynamics of microtubules in the bloodstream form, despite that in the bloodstream form both proteins appear to possess a few features that are conserved in the procyclic form. It should be noted that life cycle-specific differences in many other cellular processes, such as glucose transport [21], chromatin structure [22], maintenance of plasma membrane potential [23], cell cycle regulation [24], [25], and cell motility [5], have been well recognized in trypanosomes previously. However, the molecular basis underlying these differences between the two life cycle forms remains a mystery. Our current finding suggests that regulation of the cytoskeletal microtubule corset likely also differs between the two life cycle stages.

Another distinct but contrasting phenotype caused by TbKIN-C and TbKIN-D RNAi between the two life cycle forms is the production of cells with an enlarged flagellar pocket in the bloodstream form (Fig. 7), but not in the procylic cells [15], [16]. The enlarged flagellar pocket appeared to harbor multiple flagellar axonemes that each associated with a normal paraflagellar rod (Fig. 7), which is usually only detected in the flagellum that has exited the flagellar pocket. Moreover, multiple flagellar axoneme-like structures were found inside the cytoplasm near the flagellar pocket (Fig. 7). These observations suggest that RNAi of TbKIN-C and TbKIN-D disrupted flagellar pocket morphogenesis in the bloodstream form. However, it is not clear whether the two kinesins are directly involved in flagellar pocket morphogenesis. Rather, the abnormal flagellar pocket could be a result of the distorted cell morphology. Moreover, the enlarged flagellar pocket in the RNAi cells also suggests that there may be defects in endocytosis, which requires further investigation.

Although RNAi of TbKIN-C and TbKIN-D caused distinct phenotypes in the two stages of life cycle, knockdown of both kinesins disrupted basal body segregation and cytokinesis in both the procyclic and bloodstream forms (Fig. 4; [15], [16]). Basal body segregation in trypanosomes is generally regarded as the first cytoskeletal event of the cell division cycle and is known to play a dominant role in control of cytokinesis in the procyclic form of *T.* *brucei* [26]. Our finding tempts to suggest that basal body segregation, even over a relatively short distance during the cell cycle in the bloodstream form, also plays an important role in driving cytokinesis progression in the bloodstream form (Fig. 4), similar to its essential role in the procyclic form where the duplicated basal bodies are farther separated than that in the bloodstream form. Nevertheless, segregation of the duplicated basal bodies in the bloodstream form is apparently not the major driving force for cytokinesis as previous reports have demonstrated that bloodstream trypanosomes that are deficient in mitosis but not in basal body duplication and/or segregation are still arrested in cytokinesis [24], [25], [27]. However, RNAi of either TbKIN-C or TbKIN-D in the bloodstream form appeared to exert little effect on mitosis (Figs. 3 and 4), thus suggesting that the cytokinesis arrest of TbKIN-C and TbKIN-D RNAi cells was not attributed to any mitotic defects.

An intriguing observation made in this paper is the interdependence of TbKIN-C and TbKIN-D for maintaining protein stability in both life cycle forms (Figs. 8 and 9). Given that the two kinesins form a complex (Fig. 1) and that both kinesins are very stable when in a complex in wild-type cells (Fig. 9), this observation suggests that formation of TbKIN-C/TbKIN-D complex prevents the two kinesins from being degraded by the 26S proteasome. It also suggests that TbKIN-C and TbKIN-D homodimers are either not formed in trypanosome cells or are not stable. Many kinesins are known to perform their cellular functions as homodimer or heterodimer [28], and homodimerization is necessary for the processivity of kinesin motors [29]. To maintain the processivity of heterodimeric kinesins, it is thought that the two different kinesins coordinate their mechanochemical cycles [30]. For example, ATP binding by one kinesin motor triggers the release of ADP from the other kinesin motor [31]. In another mechanism, an unprocessive kinesin can be turned processive through heterodimerization with a processive kinesin partner [32]. Our previous results have demonstrated that TbKIN-C and TbKIN-D also form a heterodimer via interactions between the C-terminal coiled-coil motifs of the two kinesins [15], [16]. Although both TbKIN-C and TbKIN-D possess ATPase activity [15], [16], it is not clear whether formation of TbKIN-C/TbKIN-D heterodimer maintains the processivity by coordinating their mechanochemical cycles as observed in other heterodimeric kinesins [31]. Nevertheless, our current data argues that formation of TbKIN-C/TbKIN-D heterodimer is necessary for maintaining the stability of both kinesins, which represents a new mechanism for heterodimerization of kinesins in eukaryotes, i.e. for stabilization of the kinesins in the heterodimeric kinesin complex.

## Materials and Methods

### Trypanosome Cell Culture and RNA Interference

The bloodstream-form Single Marker (SM) cell line [33] was cultured in HMI-9 medium supplemented with 10% fetal bovine serum (Atlanta Biologicals, Inc) and 15 µg/ml G418 in a 37°C incubator supplied with 5% CO_2_. The wild-type 221 strain [34] was cultivated in HMI-9 medium containing 10% fetal bovine serum at 37°C and 5% CO_2_. The procyclic cell lines harboring the RNAi construct against TbKIN-C or TbKIN-D were cultured in SDM-79 medium supplemented with 10% fetal bovine serum, 50 µg/ml hygromycin, 15 µg/ml G418, and 2.5 µg/ml phleomycin at 27°C.

The RNAi plasmids, pZJM-TbKIN-C and pZJM-TbKIN-D, have been described previously [15], [16]. Transfection of bloodstream SM cell line by electroporation was carried out according to our published procedures [27], [35]. Briefly, after electroporation, the cells were resuspended in 24 ml HMI-9 medium containing 15 µg/ml G418 and cultured in a 24-well plate. After 24 hours, 2.5 µg/ml phleomycin was added. Successful transfectants were further cloned by limiting dilution in a 96-well plate. To induce RNAi, the monoclonal cell line was incubated with 1.0 µg/ml tetracycline.

### Quantitative RT-PCR

Total RNA was purified from *T. brucei* cells with the TRIzol reagent (Invitrogen) and treated with DNase I to remove any contaminated DNA. First-strand cDNA was then synthesized with MMLV reverse transcriptase (Promega), and real-time RCR was carried out using the SYBR green PCR master mix (Applied Biosystems) on the CFX Real-Time PCR System (Bio-Rad). Actin gene was used as the control. Three replicates of PCR reaction were run simultaneously in the Real-Time PCR machine.

### Epitope Tagging of Endogenous TbKIN-C and TbKIN-D in the Bloodstream Form

For subcellular localization of TbKIN-C and TbKIN-D in the bloodstream form, wild-type 221 cells were electroporated with pC-TbKIN-C-EYFP-NEO and pC-TbKIN-D-EYFP-NEO and selected by adding 15 µg/ml G418 to the culture medium. Localization of TbKIN-C-EYFP and TbKIN-D-EYFP in intact cells and in the cytoskeleton of trypanosome cells was visualized and captured using a fluorescence microscope. The two endogenous tagging constructs have been described previously [15], [16].

To co-express TbKIN-D-PTP and TbKIN-C-3HA in the bloodstream cells for co-immunoprecipitation, wild-type 221 cells were first transfected with pC-TbKIN-D-PTP-NEO and selected with 15 µg/ml G418. The clonal cell line was obtained by limiting dilution and was transfected with pC-TbKIN-C-3HA-BSD. The double transfectanted cells were selected with 10 µg/ml blasticidin in addition to 15 µg/ml G418.

To detect the level of proteins upon RNAi induction, TbKIN-C RNAi cell line and TbKIN-D RNAi cell line were transfected with plasmid pC-TbKIN-C-3HA-BSD and pC-TbKIN-D-3HA-BSD, respectively. Transfectants were selected under 10 µg/ml blasticidin. Additionally, to monitor the expression of TbKIN-C in TbKIN-D RNAi cells and TbKIN-D in TbKIN-C RNAi cells in both the bloodstream and procyclic forms, pC-TbKIN-C-3HA-BSD or pC-TbKIN-D-3HA-BSD was electroporated into bloodstream SM cell line harboring pZJM-TbKIN-D RNAi construct or pZJM-TbKIN-C RNAi construct, respectively, and procyclic 29–13 cell line harboring pZJM-KIN-D or pZJM-TbKIN-C, respectively.

In all the endogenous tagging experiments described above, correct *in situ* tagging of one of the two *TbKIN-C* or *TbKIN-D* alleles was confirmed by PCR and subsequent sequencing of the PCR fragment. Correct tagging of the protein was also verified by Western blot with anti-EYFP mAb (JL-8 clone, Clontech), anti-HA mAb (Sigma-Aldrich), and anti-Protein A mAb (Sigma-Aldrich) for EYFP-, HA-, and PTP-fusion proteins, respectively.

### Flow Cytometry

Flow cytometry analysis of propidium iodide-stained bloodstream-form cells was carried out as previously described [36]. Cells were fixed in ethanol and re-suspended in PBS containing 10 µg/ml DNase-free RNase and 20 µg/ml propidium iodide. The DNA content of propidium iodide-stained cells was analyzed with a fluorescence-activated cell sorting scan (FACScan) analytical flow cytometer (BD Biosciences). The percentage of cells in each phase of the cell cycle (G1, S, and G2/M) was determined by the ModFit LT V3.0 software (BD Biosciences).

### Immunofluorescence Microscopy

Cells were washed three times with PBS, fixed in 4% paraformaldehyde, and adhered to poly-L-Lysine treated coverslips. The cells on the coverslip were then incubated with primary antibodies for 1 hr at room temperature. The following antibodies were used in the present study: YL 1/2 mAb for tyrosinated α-tubulin (1∶400 dilution) [37]; L8C4 mAb for the flagellum (1∶50 dilution) [7]; L3B2 mAb for the FAZ filament (1∶50 dilution) [7]; FITC-conjugated anti-HA antibody (1∶400) (Sigma-Aldrich). After incubating with YL 1/2 antibody, cells were washed three times with wash buffer (0.1% Triton X-100 in PBS), and then incubated with FITC-conjugated anti-rat IgG at room temperature for 1 hr. For immunostaining with FITC-conjugated anti-HA antibody, cells were washed three times with wash buffer and mounted. All slides were mounted in VectaShield mounting medium (Vector Labs) containing DAPI and examined under an inverted microscope (Model IX71, Olympus) equipped with a cooled CCD camera (Model Orca-ER, Hamamatsu) and a PlanApo N 60× 1.42-NA DIC objective. Images were acquired and processed with the Slidebook5 software (Intelligent Imaging Innovations, Inc).

### Preparation of Thin Sections of Trypanosomes and Transmission Electron Microscopy

Preparation of thin sections of trypanosome cells for electron microscopy follows the published procedures [15], [16]. Briefly, control cells and RNAi cells after tetracycline induction for 2 days were fixed in glutaraldehyde, treated with Millonig’s buffer, and incubated with 2% OsO_4_ at 4°C for 60 min. Cells were then dehydrated with ethanol and embedded in resin. The fixed cells were embeded in BEEM capsules before polymerizing overnight at 70°C. The 120 nm thin sections were cut using a Leica Ultracut-R microtome and a diamond knife (Daitome-U.S.), placed on 150 mesh copper grids (EMS), and stained with uranyl acetate. The thin sections were then rinsed with water and incubated with Renold’s lead citrate for 5 min. Grids were imaged using a JEOL 1400 TEM at 60 kv and captured with a Gatan CCD camera.

### Co-immunoprecipitation

Bloodstream-form 221 cells co-expressing TbKIN-D-PTP and TbKIN-C-3HA or expressing TbKIN-D-PTP alone were harvested, lysed in trypanosome immunoprecipitation (IP) buffer (25 mM Tris-Cl, pH7.6, 100 mM NaCl, 1 mM DTT, 1% Nonidet P-40, and protease inhibitor cocktail), cleared by centrifugation, and incubated with 20 µl IgG sepharose beads (GE HealthCare Life Sciences) at 4°C for 2 hrs. To dissociate the kinesin proteins from microtubules, the cells were lysed in IP buffer containing 1 mM ATP and 500 mM NaCl, cleared by centrifugation, and then incubated with IgG sepharose beads. The beads were washed six times with the IP buffer, re-suspended in 10% SDS to elute the proteins precipitated with IgG beads, and the supernatant were loaded onto a SDS-PAGE gel. Western blot was performed with anti-HA mAb (Sigma-Aldrich) to detect co-precipitated TbKIN-C-3HA, with anti-Protein A mAb (Sigma-Aldrich) to detect TbKIN-D-PTP, and with anti-tubulin mAb (Sigma-Aldrich) to detect the tubulins.

### Protein Stability Assay

To examine the level of TbKIN-C protein in TbKIN-D RNAi cells and the level of TbKIN-D protein in TbKIN-C RNAi cells, bloodstream cells harboring TbKIN-D RNAi and pC-TbKIN-C-3HA- BSD constructs or TbKIN-C RNAi and pC-TbKIN-D-3HA-BSD constructs were induced with tetracycline for 3 or 4 days, and cells were collected every day for western blot with anti-HA antibody. For MG132 treatment, the bloodstream cells harboring TbKIN-D RNAi and pC-TbKIN-C-3HA-BSD constructs and the cells harboring TbKIN-C RNAi and pC-TbKIN-D-3HA-BSD constructs were induced with tetracycline for 2 and 3 days, respectively. Both cell lines were split to two identical cell samples, and one sample was treated with 25 µm MG132 for 6 hrs. Cells were lysated and then immuboblotted with anti-HA antibody to detect 3HA-tagged TbKIN-C and TbKIN-D. The same blots were stripped and re-probed with anti-TbPSA6 antibody to detect the α6 subunit of the 26S proteasome as the loading control.

The procyclic cells harboring TbKIN-D RNAi and pC-TbKIN-C-3HA-BSD constructs or TbKIN-C RNAi and pC-TbKIN-D-3HA-BSD constructs were induced with tetracycline for 2 days and then split to two identical cell samples, one of which was treated with 50 µm MG132 for 8 hrs. Both cell lines and the uninduced control cells were collected for western blot with anti-HA antibody for TbKIN-C -3HA and TbKIN-D-3HA proteins. The same blot was re-probed with anti-TbPSA6 antibody as the loading control.

To measure the half-life of TbKIN-C and TbKIN-D, the procyclic cells expressing endogenously 3HA-tagged TbKIN-C and TbKIN-D were treated with 100 µg/ml cycloheximide for 12 hours in SDM-79 medium containing 10% fetal bovine serum and appropriate antibiotics at 27°C. As a positive control, the procyclic cell line expressing 3HA-tagged TbAUK1 was also treated with 100 µg/ml cycloheximide for 12 hours. TbAUK1 is known to be degraded by the 26S proteasome [38]. After cycloheximide treatment, equal number of cells was collected every 4 hours. Cells were lysed in 1x SDS-PAGE sampling buffer, separated in SDS-PAGE, transferred onto a PVDF membrane, and immunoblotted with anti-HA antibody. The same blots were stripped and re-probed with anti-TbPSA6 antibody to detect the α6 subunit of the 26S proteasome, which is known to be stable and not degraded by the proteasome [39].
